# Serum proteome profiling reveals SOX3 as a candidate prognostic marker for gastric cancer

**DOI:** 10.1111/jcmm.15326

**Published:** 2020-05-04

**Authors:** Jiajia Shen, Jing Zhai, Xinqian Wu, Guiping Xie, Lizong Shen

**Affiliations:** ^1^ Division of Gastrointestinal Surgery Department of General Surgery First Affiliated Hospital Nanjing Medical University Nanjing China; ^2^ Department of Surgical Oncology Jiangsu Province Hospital of Chinese Medicine Affiliated Hospital of Nanjing University of Chinese Medicine Nanjing China

**Keywords:** biomarker, gastric cancer, prognosis, proteome profiling, SOX3

## Abstract

Searching for the novel tumour biomarkers is pressing for gastric cancer diagnostication and prognostication. The serum specimens from patients diagnosed with locally advanced gastric carcinoma before operation and 4 week after surgery were collected, respectively, and serum proteome profiling was conducted by liquid chromatography–mass spectrometry (MS)/MS. Fifty‐five proteins were identified to be up‐regulated and 16 proteins were down‐regulated, and these differentially expressed proteins participated in various biological processes. Serum levels of SOX3, one of down‐regulated proteins, in stomach cancer patients were higher than in healthy controls. SOX3 levels in cancer tissues were remarkably related to tumour differentiation, lymph node metastasis, primary tumour invasion and pTNM (pathological TNM) stage. Analysis with The Cancer Genome Atlas database indicated that SOX3 level and pTNM stage were the independent risk factors for the patient survival and that the overall survival was negatively associated with the SOX3 levels. Loss‐of‐function showed that SOX3 promoted gastric cancer cell invasion and migration in vitro and in vivo. SOX3 silence inhibits the expression of MMP9, and SOX3 is responsible for MMP9 expression transcriptionally. Our study highlights the potentiality of the paired pre‐ and post‐operation serum proteome signatures for the detection of biomarkers and reveals that SOX3 may serve as a candidate prognosis marker for gastric cancer.

## 1 INTRODUCTION

Gastric carcinoma remains one of the most prevalent life‐threatening cancers worldwide. There were more than 1 000 000 new gastric cancer cases and nearly 800 000 cancer‐associated deaths in the world in 2018 according to estimates from the International Agency for Research on Cancer.^1^, ^2^ In China, it was estimated that there were 410 400 new cases of stomach cancer and 293 800 deaths related to stomach cancer in 2014.^3^, ^4^ Radical gastrectomy with D2 lymph node dissection is the only potentially curative treatment for locally advanced disease. However, about half of patients undergoing curative surgery eventually relapse, and nearly two‐thirds of cases present with incurable advanced or metastatic disease when they are diagnosed. The serial advances in post‐operative adjuvant chemotherapy, neoadjuvant chemotherapy or perioperative chemotherapy have reshaped the treatment strategies for gastric cancer.^5^ Perioperative epirubicin/cisplatin/5‐fluorouracil chemotherapy improved significantly the 5‐year overall survival (OS) from 23% to 36%.^6^ Recently, perioperative FLOT regimen (docetaxel/oxaliplatin/5‐fluorouracil) was established as the new standard of care for resectable adenocarcinoma of the gastroesophageal junction and the stomach.^7^

However, no appropriate biomarkers to refine treatment selection and to monitor treatment response have been established in this disease to date, which limits further improvement of these combination regimens for gastric cancer patients towards the goal of precise medicine. Gastric cancer still has no validated and well‐recognized tumour biomarkers that are applied to clinical management and prognostication. Tumour biomarkers represent substances that are characteristic of malignant tumour cells, or are disproportionately produced by malignant tumour cells, or are produced by the host's stimulating response to tumours. Candidate tumour markers may involve distinguishing alterations in tumour genomics, epigenomics, gene expression and transcriptomic profiles, protein expression, cellular composition of the microenvironment, and vasculature.^8^, ^9^ Tumour markers should reflect tumour occurrence and development, predict the response to specific treatment and estimate survival.^8^ Serum‐based biomarkers, including carcinoembryonic antigen, cancer antigen 19‐9, CA 125 and CA724 have been used clinically to monitor gastric cancer, especially to detect disease recurrence after radical surgery.^10^, ^11^ However, they lack specificity, display low sensitivity and do not fulfil the clinical practice.^12^ Thus, it is necessary and pressing to search for novel tumour biomarkers for gastric cancer diagnostication and prognostication. Tumour markers may exist in tumour tissues, body fluids and excreta of patients. Tumour marker should be detected by immunological, biological or chemical methods. Circulating biomarkers are convenient to be assayed and to be monitored dynamically. We conceive that the expression of serum proteins in gastric cancer patients may be altered after radical operation, and some of the differentially expressed proteins (DEPs) may be directly associated with cancer clearance and some of the DEPs may be candidate circulating biomarkers for gastric cancer.

To this end, we collected the serum specimens from six patients diagnosed with locally advanced gastric carcinoma before operation and 4 weeks after surgery, respectively, and conducted serum proteome profiling with these paired specimens by liquid chromatography–mass spectrometry (LC‐MS/MS) in the present study. We found that 55 proteins were up‐regulated and 16 proteins were down‐regulated in post‐operative serums compared with preoperative serums. Among these down‐regulated 16 proteins, the level of sex‐determining region Y‐related high‐mobility group (HMG) box 3 (SOX3) in the post‐operative serums was less than 50% of that in preoperative ones. The validation study showed that SOX3 is overexpressed in cancer tissues, and its levels are associated with poor outcomes for stomach cancer, and that SOX3 promotes gastric cancer cell invasion and migration through MMP9. Our preliminary results demonstrated for the first time that SOX3 expression in the serums and in the tumour tissues may serve as a candidate marker for prognosis and outcomes of gastric carcinoma patients.

## 2 MATERIALS AND METHODS

### 2.1 Serum and tissue specimens of patients with gastric cancer

A total of 60 patients with gastric cancer from January 2017 to December 2018 at the Department of General Surgery, First Affiliated Hospital, Nanjing Medical University, were enrolled in our study retrospectively. These patients included five cases of early gastric carcinoma and 55 cases of locally advanced gastric carcinoma, and they underwent curative radical gastrectomy with D2 lymphadenectomy. These patients did not receive chemotherapy or radiotherapy before operation. The pre‐treatment serum specimens were collected preoperatively from these patients, and 60 cases of age‐ and sex‐matched healthy donors were also enrolled as a control group. The post‐operation serum specimens were collected on 30 days after operation and before adjuvant chemotherapy in these patients, and these patients had no severe surgical complications. The gastric cancer tissues and the corresponding normal mucosa tissues at least 5 cm from the outer tumour margin were collected from all patients immediately after resection. All the sample studies were performed following written consent according to an established protocol approved by the Institutional Review Board of Nanjing Medical University. This study was also in accordance with the Declaration of Helsinki.

### 2.2 Cell culture

Human gastric adenocarcinoma cell lines, AGS (ATCC, VA, USA) and MKN45 (CBTCCCAS, Shanghai, China), were cultured in the complete DMEM (ATCC) supplemented with 10% foetal bovine serum (Hyclone, Logan, UT, USA).

### 2.3 Protein extraction, trypsin digestion, TMT/iTRAQ labelling and HPLC fractionation

Protein extraction, trypsin digestion, TMT/isobaric tags for relative and absolute quantitation (iTRAQ) labelling and HPLC fractionation were performed according to the manufacturer's instructions. For details, see Supplementary Materials and Methods.

### 2.4 LC‐MS/MS analysis

Liquid chromatography–mass spectrometry/MS analysis was performed according to the manufacturer's instructions. For details, see Supplementary Materials and Methods.

### 2.5 Western blot assay

Protein expression levels of the indicated molecules were assayed using the Western blotting.^13^ The antibodies used for the assays were as following: rabbit monoclonal anti‐matrix metalloproteinase 2 (MMP2) antibody, rabbit monoclonal anti‐MMP7 antibody, rabbit monoclonal anti‐MMP9 antibody, rabbit monoclonal anti‐GAPDH antibody (Cell Signaling Technology, MA, USA), rabbit monoclonal anti‐β‐actin antibody (Cell Signaling Technology, MA, USA), rabbit monoclonal anti‐SOX3 antibody (Abcam, Cambridge, UK).

### 2.6 Enzyme‐linked immunosorbent assay

The ELISA (Invitrogen, CA, USA) was applied in measuring SOX3 in the patient serums. Each experiment was repeated at least three times.

### 2.7 Cell proliferation assay

Cell proliferation was analysed using a Cell Counting Kit‐8 (CCK‐8) assay (Dojindo, Japan) according to the manufacturer's protocol. The results were plotted as mean ± SE of three separate experiments for each experimental condition.

### 2.8 Immunohistochemistry

SOX3 expression in gastric tissues was detected by immunohistochemistry (IHC). A rabbit monoclonal anti‐SOX3 antibody (Abcam, Cambridge, UK) was used. Immunohistochemistry was performed on paraffin‐embedded formalin‐fixed tissues according to standard protocols.

### 2.9 Immunofluorescence

The section thickness of paraffin‐embedded samples is 4 mm. In the 0.01 mol/L citric acid buffer, the antigen was extracted from the pressure cooker in 20 minutes and then sectioning at room temperature for 2 hours in the phosphate buffer saline (PBS) containing 10% bovine serum albumin. After blocking, samples were incubated with primary antibodies specific for mouse anti‐α‐smooth muscle actin (anti‐α‐SMA) (1:100), rabbit‐anti‐SOX3 (1:200) overnight at 4℃. Incubation of Rhodamine Red‐X (RRX) goat antimouse immunoglobulin G (IgG) (H + L) and FITC‐AffiniPure goat anti‐rabbit IgG (H + L) (Jackson, PA, USA) was carried out for 1 hour at room temperature. Cell nuclei were counterstained with DAPI (Sigma‐Aldrich, MO, USA). Images were acquired on a Zeiss LSM510 confocal microscope (Oberkochen, Germany).

### 2.10 RNA interference analysis and cell transfection

Lentiviruses carrying SOX3 short hairpin RNA (shRNA) were constructed by Shanghai GenePharma Co., Ltd. (Shanghai, China). The transduction was performed according to the manufacturer's protocol. The shRNA targeting sequences were CCGGCGGCGCTCAGAGCTACATGAACTCGAGTTCATGTAGCTCTGAGCGCCGTTTTT. The knockdown efficiency was verified by Western blotting assay. Cell transfection was performed using Lipofectamine 3000 (Invitrogen, CA, USA).

### 2.11 Spheroid cell invasion assay

Spheroid cell invasion assay was carried out using 96 Well 3D Spheroid BME Cell Invasion Reagent Kit (Trevigen, MD, USA). For the details, see Supplementary Materials and Methods.

### 2.12 Establishment of patient‐derived xenograft model in zebrafish (zPDX)

MKN45 cells were injected into zebrafish embryos according to our previous report.^14^ The number of metastatic tumour cells was analysed using fluorescence confocal microscopy. Statistical analysis can be carried out by synthesizing pictures of different colours at the same level.

### 2.13 Chromatin immunoprecipitation

Chromatin immunoprecipitation assays were performed with the SimpleCHIP^®^ Enzymatic Chromatin IP Kit (Cell Signaling Technology, MA, USA) following manufacturers’ recommended protocols. Cell lysates of MKN45 (4 × 10^7^) were prepared, and chromatin fragments were fragmented to an average size of 150‐900 bp by microcapsule nuclease, and enriched with magnetic beads coated with SOX3 antibodies or isotype IgG. Then, the concentrated sample was crosslinked with the input DNA, and the DNA was purified with sodium chloride and protease K. Finally, the specific sequences from immunoprecipitated and input DNA were determined by RT‐qPCR for the upstream of MMP9 promoter region. Three primer pairs of MMP9 promoter region used in RT‐qPCR analyses were listed as follows: CTTTCCCTTGGCTGACCACT (forward primer) and AAACTGCAGAGCTTGTGGGA (reverse primer).

### 2.14 Statistical analysis

Data are expressed as mean ± SE. In experiments involving protein expression, the data were representative of three independent experiments. The associations between the protein levels and various clinicopathological parameters were analysed with Pearson's chi‐square test. Quantitative data were compared between the control and treatment groups by analysis of variance. All analyses were performed with SPSS software (version 19.0; SPSS Inc, Chicago, IL, USA). Values of *P* < .05 were considered to indicate statistical significance.

## 3 RESULTS

### 3.1 Serum proteome profiling reveals several differential expressed proteins, including SOX3, between pre‐ and post‐operation for locally advanced gastric cancer

**FIGURE 1:**
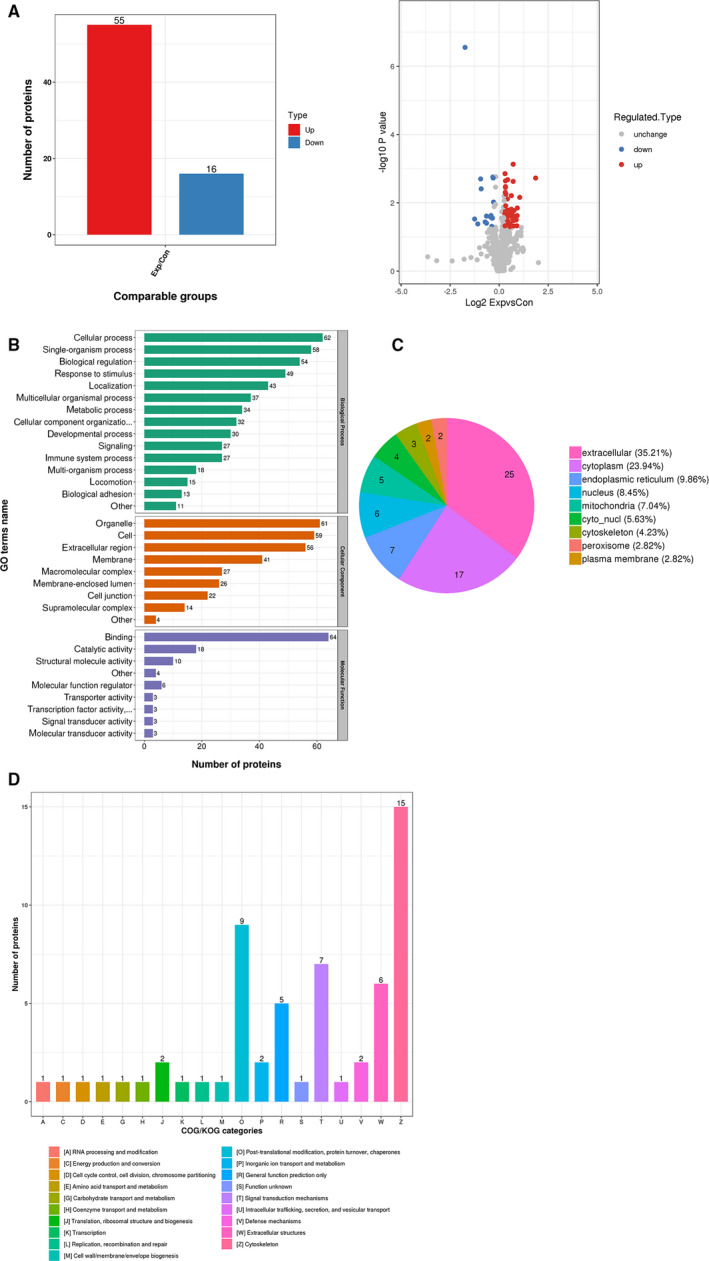
The differentially expressed proteins (DEPs) and their distribution. A, Fifty‐five proteins were up‐regulated and 16 proteins were down‐regulated in post‐operative serums (Exp) compared with preoperative serums (Con). B, Analysis with GO secondary annotations showed that these DEPs were enriched in 33 terms. C, These DEPs were mainly located in extracellular space (35.21%) and cytoplasm (23.94%). D, Analysis of COG/KOG indicated that these DEPs mainly participated in cytoskeleton formation

Six patients diagnosed with locally advanced gastric adenocarcinoma were enrolled in serum proteome profiling assay, and this assay identified 803 proteins, and 657 of them contained quantitative information. Fifty‐five proteins were up‐regulated and 16 proteins were down‐regulated in post‐operative serums (Exp) compared with preoperative serums (Con) when 1.2 times was used as the differential expression threshold and *P* < .05 by *t* test was used as the significance threshold (Figure 1A). For the distribution of these 71 DEPs, analysis with gene ontology (GO) secondary annotations showed that these DEPs were enriched in 33 terms, including 15 in the category of ‘biological processes’, nine in ‘cell components’ and nine in ‘molecular functions’ (Figure 1B). These DEPs were mainly located in extracellular space (35.21%) and cytoplasm (23.94%) (Figure 1C) predicted with Wolfpsort. Analysis of COG/KOG (clusters of orthologous groups of proteins/eukaryotic orthologous groups of proteins) functional classifications indicated that these DEPs mainly participated in cytoskeleton formation (Figure 1D).

Furthermore, the enrichment analyses of DEPs at GO classification, KEGG (encyclopedia of genes and genomes) pathway and protein domains were carried out to determine whether these 71 DEPs had a significant enrichment trend in some specific functional types. Analysis of GO classification, including cellular component, biological process and molecular function, showed that these DEPs were mainly enriched for regulation of cell migration (Figure S1Aa), cell junction (Figure S1Ab) and actin binding (Figure S1Ac). KEGG pathway analysis revealed that these DGPs were abundant in biological pathways, including influenza A, antigen processing and presentation, leucocyte transendothelial migration (Figure S1B). Protein domains refer to certain components that appear repeatedly in various protein molecules and have similar sequences, structures and functions. The enrichment and distribution of these DEPs in protein domain classification were mainly concentrated in PH domain‐like, calponin homology domain, ubiquitin‐related domain (Figure S1C). These results collectively suggest that the serum proteome profiling in gastric cancer patients changes after operation, and these DEPs participate in various biological processes, which may be associated with tumour elimination and/or surgical stress.

**FIGURE 2:**
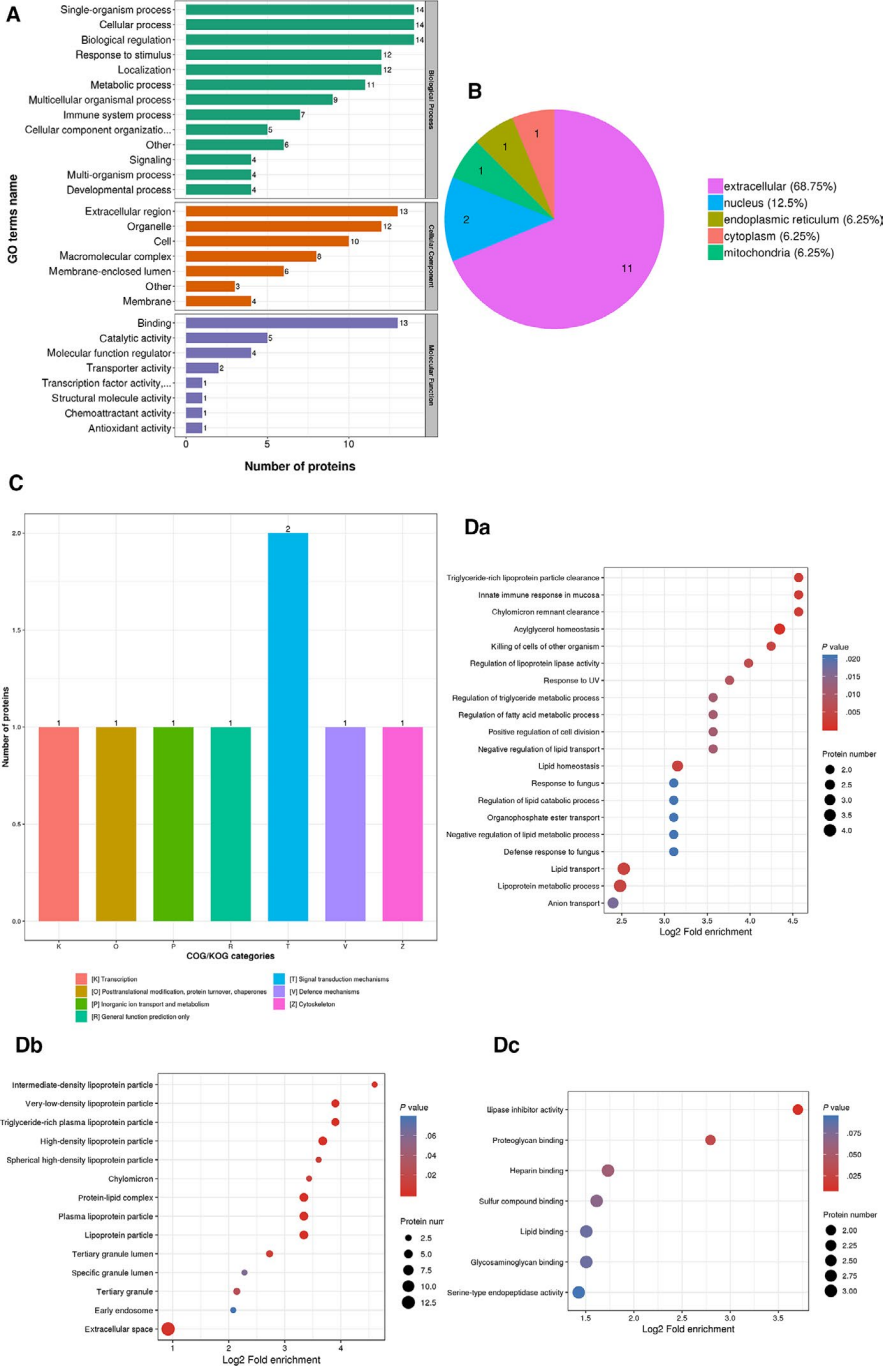
Analysis of the 16 down‐regulated proteins. A, The 16 differentially expressed proteins (DEPs) were enriched in 28 terms. B, The 16 DEPs were mainly located in extracellular space (68.75%). C, The 16 DEPs distributed in seven categories functionally. D, The 16 DEPs were mainly enriched for lipid transport (Da), lipoprotein particle (Db) and lipase inhibitor activity (Dc)

**FIGURE 3:**
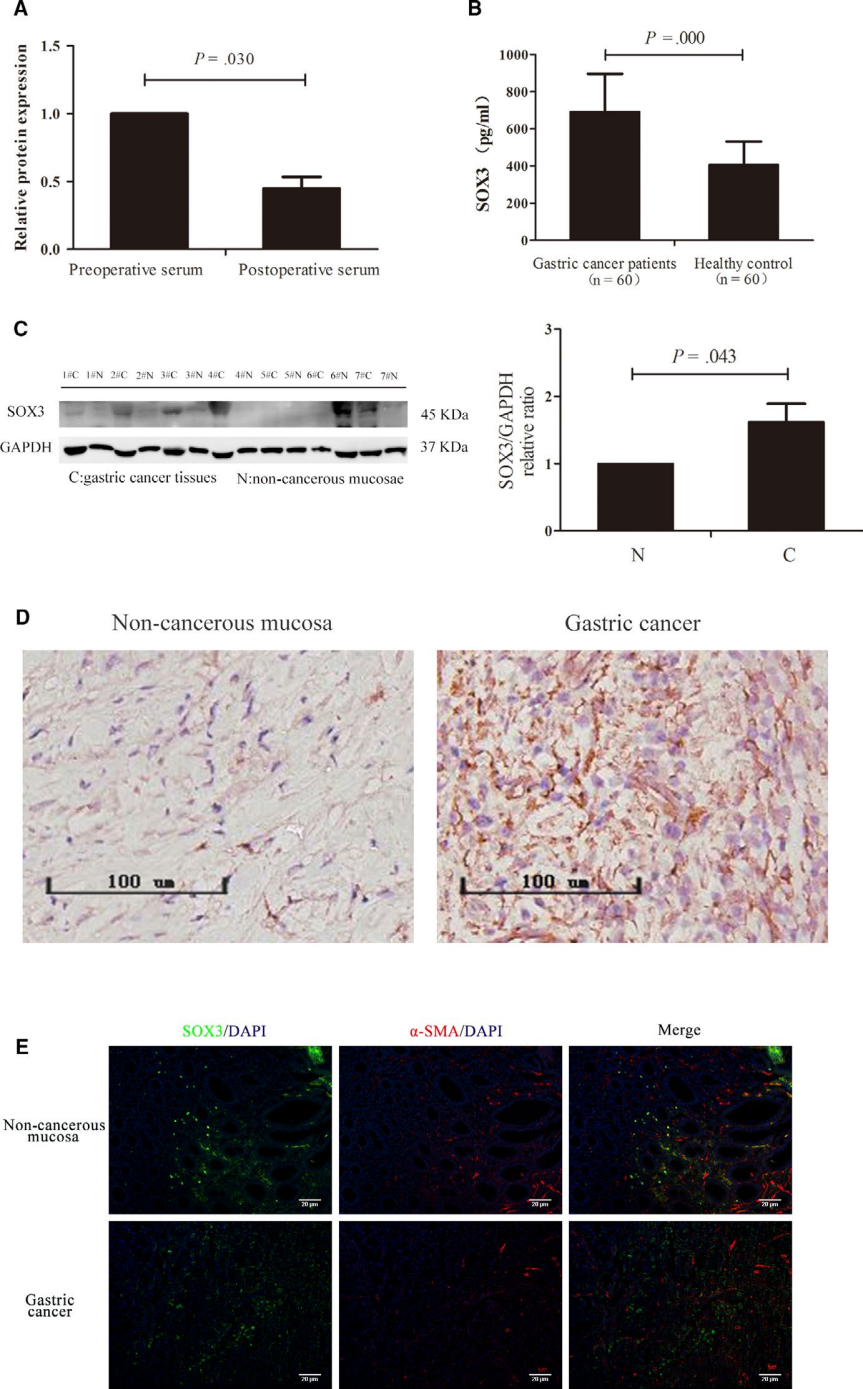
SOX3 expression in gastric cancer tissues. A, ELISA assay with 60 gastric cancer cases showed SOX3 level in the post‐operative serums was less than in preoperative ones (*P* = .030). B, Serum SOX3 levels in gastric cancer patients was higher than in healthy controls (*P* = .000). C and D, SOX3 levels in gastric tumour tissues were higher than that in corresponding non‐cancerous mucosae. E, SOX3 was expressed in tumour parenchyma rather than tumour stroma by immunofluorescence assay (green for SOX3 and red for α‐SMA)

Among these DEPs, 16 down‐regulated proteins were of great interest, as they may be produced by tumour tissues and may be served as tumour markers. In GO secondary annotations, these 16 DEPs were enriched in 28 terms, including 13 in the category of ‘biological processes’, seven in ‘cell components’ and eight in ‘molecular functions’ (Figure 2A). They were mainly located in extracellular space (68.75%) (Figure 2B). These 16 proteins distributed in seven categories functionally according to analysis of COG/KOG functional classifications (Figure 2C), and they were mainly enriched for lipid transport (Figure 2Da), lipoprotein particle (Figure 2Db) and lipase inhibitor activity (Figure 2Dc). In these 16 proteins, we paid much attention to SOX3, and its level in the post‐operative serums was less than 50% of that in preoperative ones, which was confirmed in 60 cases of gastric cancer by ELISA assay (Figure 3A; *P* = .030), indicating that SOX3 may be associated with gastric cancer. However, the role of SOX3 in this disease remains uncertain so far. Therefore, we conducted subsequent studies on SOX3.

### 3.2 SOX3 is overexpressed in gastric cancer tissues and is associated with poor outcomes for gastric cancer

To validate the results of SOX3 in LC‐MS/MS, we assayed the SOX3 levels in the pre‐treatment serums from patients with gastric cancer and healthy controls, respectively, and found that the SOX3 level in patients (693.23 ± 26.21 pg/mL, N = 60) was higher than that in healthy controls (406.45 ± 16.14 pg/mL, N = 60) (Figure 3B; *P* = .000). Furthermore, immunoblotting assays (Figure 3C) and IHC studies (Figure 3D) showed that the SOX3 levels in gastric cancer tissues were higher than that in corresponding non‐cancerous mucosae (*P* = .043). We next detected SOX3 location in tumour tissues using immunofluorescence and ascertained that SOX3 was expressed in tumour parenchyma rather than tumour stroma (Figure 3E). These results demonstrated that SOX3 was differentially overexpressed in gastric cancer cells.

**FIGURE 4:**
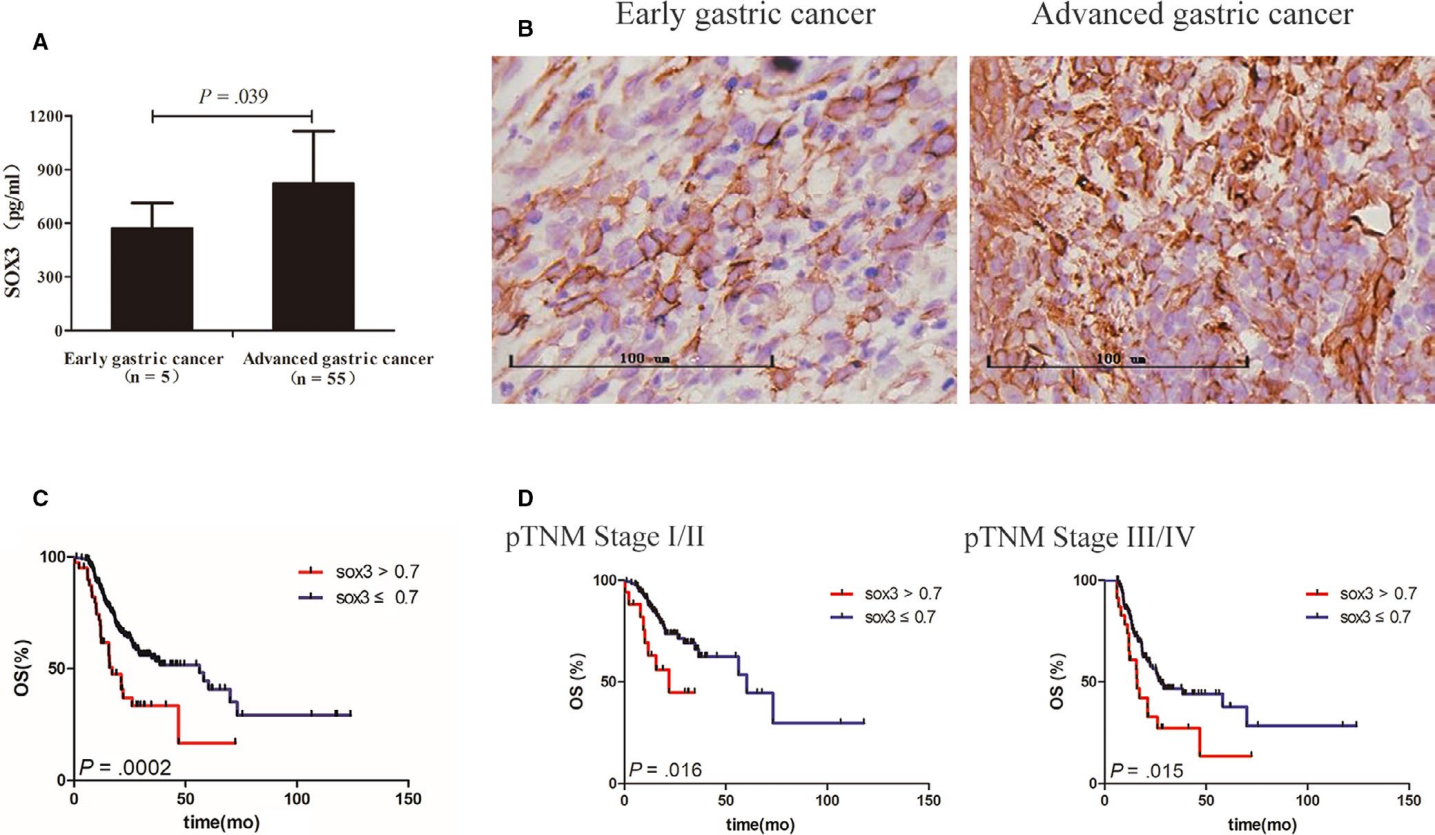
Clinical relevance of SOX3 in gastric cancer. A, Serum SOX3 level was higher in advanced cancer patients than in early cancer patients (*P* = .039). B, SOX was highly expressed in advanced gastric cancer tissues compared with that in early tumour. C, The overall survival (OS) of 304 gastric cancer patients was negatively associated with the SOX3 levels in tumour tissue (*P* = .0002). D, Stratified analyses indicated that the SOX3 levels in tumour tissues were correlated with the OSs in both patients of pTNM stage Ⅰ/Ⅱ (*P* = .016) and stage Ⅲ/Ⅳ (*P* = .015) conversely

We then evaluated the clinical relevance of the serum SOX3 levels in gastric cancer patients. We found that the serum SOX3 levels were remarkably associated with tumour differentiation (*P* < .001), lymph node metastasis (*P* = .002), primary tumour invasion (*P* = .027) and pTNM stage (*P* < .001) (Table 1). The serum SOX3 level was also much higher in advanced cancer patients (824.42 ± 39.01 pg/mL, N = 55) than in early cancer patients (573.39 ± 62.61 pg/mL, N = 5) (Figure 4A; *P* = .039), and IHC study showed that SOX was highly expressed in advanced gastric cancer tissues compared with that in early tumour (Figure 4B).

**TABLE 1:** Clinical relevance of the serum SOX3 levels in 60 patients with gastric cancer

Clinicopathological features	N	SOX3 expression in serum		*P*‐value
		high cases (%)	low cases (%)	
Age (y)				.251
<60	21	9 (42.9)	12 (57.1)	
≥60	39	11 (28.2)	28 (71.8)	
Gender				.357
Male	34	13 (38.2)	21 (61.8)	
Female	26	7 (26.9)	19 (73.1)	
Tumour location				.115
Upper third	26	9 (34.6)	17 (65.4)	
Middle third	21	4 (19.0)	17 (81.0)	
Lower third	13	7 (53.8)	6 (46.2)	
Differentiation				<.001
Poorly	16	12 (75.0)	4 (25.0)	
Moderately	19	6 (31.6)	13 (68.4)	
Well	25	2 (8.0)	23 (92.0)	
Lymph node metastasis				.002
N0	15	0 (0.0)	15 (100.0)	
N1‐N3	45	20 (44.4)	25 (55.6)	
Primary tumour invasion				.027
T1 and T2	19	15 (78.9)	4 (21.1)	
T3 and T4	41	20 (48.8)	21 (51.2)	
Distant metastasis				
M0	54	17 (31.5)	37 (68.5)	.390
M1	6	3 (50.0)	3 (50.0)	
pTNM stage				
I/II	20	0 (0.0)	20 (100.0)	<.001
III/IV	40	20 (50.0)	20 (50.0)	

Subsequently, we further investigated the correlation between SOX3 expression in gastric cancer tissues and clinicopathological features of patients from The Cancer Genome Atlas database (www.cancergenome.nih.gov. version 2017‐09‐08), which included 304 enrolled cases diagnosed with gastric carcinoma. As shown in Table S1, the SOX3 levels in tumour tissues were remarkably correlated with primary tumour invasion (T1/2 vs T3/4, *P* = .041) and patient ages (*P* = .027). Next, we performed univariate and multivariate regression analyses for prognostic factors in the patient set. As shown in Table 2, the OS of gastric cancer patients was significantly associated with lymph node metastasis (*P* = .011), pTNM stage (*P* = .010), SOX3 level (*P* = .001) and age (*P* = .029) by univariate analysis; however, multivariate analysis indicated that pTNM stage (*P* = .008), SOX3 level (*P* < .001) and age (*P* = .002) were the independent risk factors for the patient survival.

**TABLE 2:** Univariate and multivariate analyses for prognostic factors in gastric cancer patients (TCGA database) by the Cox model

Characteristic	Univariate analysis (OS)		Multivariate analysis (OS)	
	HR (95% CI)	*P*‐value	HR (95% CI)	*P*‐value
Gender (female vs male)	1.30 (0.88‐1.93)	.186	—	—
Age (>60 y vs ≤ 60 y)	1.56 (1.05‐2.34)	.029	1.92 (1.27‐2.91)	.002
Tumour location (upper vs middle vs lower)	1.05 (0.85‐1.31)	.649	—	—
Primary tumour invasion (T1 and T2 vs T3 and T4)	1.41 (0.91‐2.19)	.121	—	—
Lymph node metastasis (N0 vs N1‐3)	1.75 (1.14‐2.68)	.011	—	—
Distant metastasis (M0 vs M1)	1.50 (0.73‐3.08)	.272	—	—
pTNM stage (I and II vs III and IV)	1.64 (1.13‐2.39)	.010	1.67 (1.14‐2.43)	.008
SOX3 (>0.7 vs ≤0.7)	2.13 (1.37‐3.31)	.001	1.85 (1.19‐2.88)	<.001

Abbreviations: HR, hazard ratio; OS, overall survival; TCGA, The Cancer Genome Atlas.

Kaplan‐Meier survival analysis of these 304 patients showed that the OS of gastric cancer patients was negatively associated with the SOX3 levels in tumour tissue (Figure 4C; *P* = .0002). Stratified analyses indicated that the SOX3 levels in tumour tissues were correlated with the OSs in both patients of pTNM stage Ⅰ/Ⅱ (*P* = .016) and stage Ⅲ/Ⅳ (*P* = .015) conversely (Figure 4D).

Thus, these results suggest that the SOX3 expression in the serums and in the tumour tissues may serve as a candidate factor for prognosis and outcomes of gastric cancer patients.

### 3.3 SOX3 promotes gastric cancer cell invasion and migration through MMP9

**FIGURE 5:**
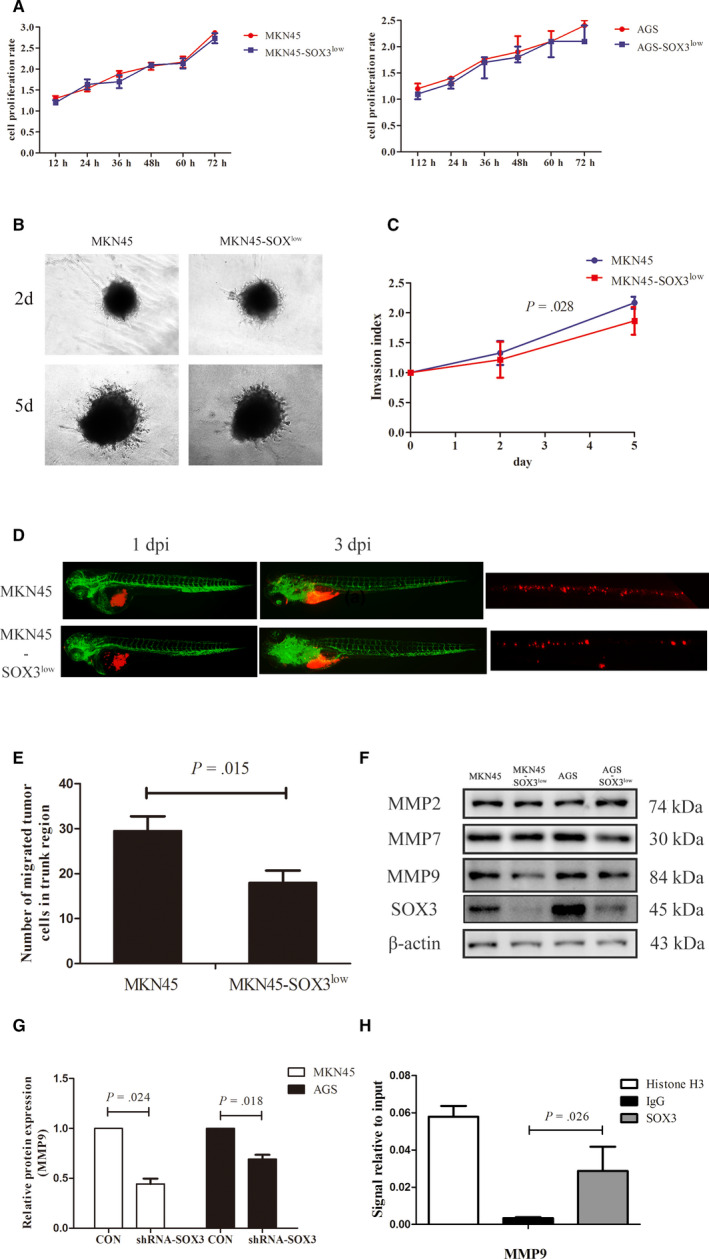
SOX3 promotes gastric cancer cell invasion and migration through MMP9. A, SOX3 silence exerted little effects on cell proliferation of MKN45 (*P* = .867) and AGS cells (*P* = .653). B and C, 3D invasion assays showed that SOX3 silence repressed MKN45 invasion significantly (*P* = .028). D and E, zPDX studies indicated that SOX3 silence repressed MKN45 migration in vivo on day 3 post‐injection (dpi) (*P* = .015) (MKN45 and MKN45‐SOX^low^ cells were shown with red fluoresce using the DiL staining). F and G, MMP9 expression was inhibited with SOX3 silence (*P* = .024 for MKN45 and *P* = .018 for AGS). H, Chromatin immunoprecipitation‐PCR assay showed SOX3 was the transcription factor responsible for MMP9 expression. zPDx, patient‐derived xenograft model in zebrafish

To investigate the effects of SOX3 on gastric cancer cells, we generated gastric adenocarcinoma cells with SOX3 silence using shRNA against SOX3, MKN45‐SOX3^low^ and AGS‐SOX3^low^. We first evaluated the influence of SOX3 on cell proliferation with CCK‐8 assays and found that SOX3 silence inhibited the proliferation of MKN45 and AGS cells but the effects did not reach remarkable significance (Figure 5A; *P* = .867 for MKN45 and *P* = .653 for AGS). We then probed the migration and invasion using in vitro three‐dimension (3D) spheroid invasion assays and in vivo zebrafish PDX (zPDX) model. 3D invasion assays showed that SOX3 silence repressed MKN45 invasion significantly (Figure 5B,C; *P* = .028), and zPDX studies indicated that SOX3 silence repressed MKN45 migration in vivo (Figure 5D,E; *P* =0.015). These results demonstrated that SOX3 promotes gastric cancer cell invasion and migration in vitro and in vivo, and it exerts little effects on cell proliferation.

We further probed the mechanisms under the effects of SOX3 on gastric cancer cell invasion and migration preliminarily. SOX3 is one of transcription factors and facilitates cell invasion and migration. We considered SOX3 expression may influence extracellular matrix in gastric cancer. Thus, we screened the expressions of MMP2, MMP7 and MMP9 in SOX3 silent cells, and found that MMP9 expression was inhibited with SOX3 silence (Figure 5F,G; *P* = .024 for MKN45 and *P* = .018 for AGS). We further evaluated the impact of SOX3 on DNA binding of MMP9 promoter region using the ChIP‐PCR assay. As shown in Figure 5H, SOX3 was the transcription factor responsible for MMP9 expression. These results revealed that MMP9 may be involved in SOX3 effects on cell invasion and migration.

## 4 DISCUSSION

Gastric carcinoma is one of highly aggressive cancers and has high mortality.^4^ There is an urgent need for appropriate markers for early diagnosis, prediction of treatment response and accurate prognosis of gastric cancer, but the current standard gastric cancer biomarkers lack availability and reliability.^15^ In the present study, we performed serum proteome profiling in the paired pre‐treatment and post‐operation serum specimens in gastric cancer patients and identified 71 DEPs. We further revealed the potentiality of SOX3 level in the serums and in the tumour tissues as a prognostic marker for gastric carcinoma patients.

Clinical proteomics is a prominent approach to discover new biomarkers for cancers.^16^ Serum‐based markers are of great interest and importance in diagnosis and monitoring of diverse diseases, including cancers. Proteins secreted by cancer tissues have a considerable chance to enter systemic circulation, and these circulating proteins may act as potential cancer markers.^10^ The blood proteome has been well recognized as a promising source of novel cancer markers.^17^ However, serum is a complicated protein mixture, and these proteins present a wide dynamic range of expression. Approximately the top ten most plentiful proteins make up 95% of the total protein content in serum. Thus, it is a great challenge to identify potential biomarkers for diseases from serum. To overcome this challenge, many state‐of‐art technologies and methods have been developed for facilitating the search for biomarkers from patient serum, including gas chromatography–mass spectrometry (GC‐MS/MS),^18^, ^19^ LC‐MS/MS,^11^, ^16^, ^20^, ^21^ surface‐enhanced laser desorption ionization time of flight mass spectrometry (SELDI‐TOF‐MS), high‐performance liquid chromatography (HPLC),^20^ MALDI‐ToF mass spectrometry,^16^ and iTRAQ.^10^, ^22^ Mass spectrometry based proteome profiling has been revealed to be potentially applicable for detection of serum proteome signature‐based biomarker for different cancer types.^15^, ^16^, ^18^, ^19^, ^20^ Based on this technique, several novel diagnostic or prognostic biomarkers have been developed for gastric cancer, including microRNA signatures,^18^ a panel of afamin, clusterin, vitamin D binding protein (VDBP) and haptoglobin,^19^ and exosomal TRIM3 ^21^ for gastric cancer screen; apolipoprotein C‐III (apoC‐III) fragment,^15^ fibrinogen α‐chain, apoA‐II and apoC‐I^20^ for diagnosis; and apoC‐III fragment^15^ for prognosis prediction.

All these current studies obtained the putative biomarkers through comparing the proteomes of serum samples between gastric cancer patients and healthy controls,^11^, ^16^, ^18^, ^20^, ^23^ or between patients with locally advanced disease and patients with metastatic cancer,^16^ or between the gastric carcinoma patients and patients with benign stomach disease.^19^, ^20^ We conceived that the DEPs between the pre‐ and post‐operative serum specimens in the same patients may be an optional and more effective source of the novel biomarkers for gastric cancer, because the DEPs may be directly related to gastric cancer tissues. Our study identified 71 DEPs, including 55 up‐regulated and 16 down‐regulated proteins, and abovementioned apoC‐III was involved. We further demonstrated SOX3 as a candidate prognostic marker for gastric cancer. These results verified our conception.

SOX family is composed of more than 20 members in vertebrates, and they mediate DNA binding via a highly conserved HMG domain. SOX members are categorized into eight groups, SOXA to SOXH,^24^ and SOX3 belongs to SOXB1 family. As transcription factors, SOX proteins are considered to participate in the regulation of specific biological processes,^25^ and SOX proteins play a critical role in development. However, SOX genes are commonly deregulated in tumours. The up‐regulation of SOX2, SOX4, SOX5, SOX8, SOX9 and SOX18 are found to be associated with poor outcome in different cancer types; however, the up‐regulation of SOX11 and SOX30 is favourable for the prognosis in other cancer types. SOX2, SOX4 and SOX5 are involved in tumorigenesis, and SOX2 is noticeably up‐regulated in chemo‐resistant cells. The SOXF family (SOX7, SOX17 and SOX18) plays a crucial role in angiogenesis or lymphangiogenesis, and SOX18 has been shown to be a potential target for antiangiogenic therapy in cancer.^25^, ^26^ There were seldom studies regarding the roles of SOX3 in cancer. Abnormal SOX3 expression has been demonstrated to induce tumorigenic transformation in chicken embryonic fibroblasts.^27^ SOX3 overexpression is involved in the pathogenesis of choriocarcinoma ^28^ and T cell lymphoma.^29^ SOX3 plays a beneficial role in tumour development and may serve as an independent risk factor of poor prognosis for oesophageal squamous cell carcinoma,^30^ and SOX3 fosters invasiveness, migration and epithelial‐mesenchymal transition in osteosarcoma cells via activating Snail1 expression transcriptionally.^31^ Recently, SOX3 is reported to maintain glioblastoma stem cells in undifferentiated state and further promote the malignant behaviour of glioblastoma cells.^32^ However, the role of SOX3 in stomach carcinoma remains uncertain. Our study demonstrated that SOX3 is highly expressed in gastric cancer tissues and is associated with poor outcomes for gastric cancer. Loss‐of‐function studies showed that SOX3 promotes gastric cancer cell invasion and migration in vitro and in vivo, and it exerts little effects on cell proliferation. SOX3 silence inhibits the expression of MMP9 rather than MMP2 and MMP7, and ChIP‐PCR assay confirms SOX3 transcription factor is responsible for MMP9 expression. Thus, MMP9 may be involved in SOX3 effects on cell invasion and migration. These results indicated SOX3 levels in serum or in tumour tissues may be a prognostic biomarker for gastric cancer patients. Of course, the other DEPs merits to be further investigated to ascertain their roles in gastric cancer.

In summary, this study highlights the potentiality of the paired pre‐ and post‐operation serum proteome signature for the detection of putative biomarkers for gastric carcinoma and reveals that SOX3 may serve as a candidate molecular marker for prognosis and outcomes of gastric cancer patients. Certainly, larger and prospective studies are needed to validate the prognostication value for gastric cancer, and further investigation is needed to elucidate the underlying mechanisms.

## CONFLICT OF INTEREST

The authors confirm that there are no conflicts of interest.

## AUTHOR CONTRIBUTIONS

L. Shen, J. Shen and J. Zhai conceived the study. J. Shen, J. Zhai and X. Wu performed the experiments and drafted the manuscript. J. Shen and J. Zhai collected all tissue samples and clinical information. G. Xie supported the experimental techniques. L. Shen reviewed the manuscript and provided financial support. All authors read and approved the final manuscript.

## Supporting information

Fig S1

Table S1

Supplementary Material

## Data Availability

The data sets used and/or analysed during the current study are available from the corresponding author on reasonable request.
