# Supplementary figures and images for: Serum proteome profiling reveals SOX3 as a candidate prognostic marker for gastric cancer
Source: J Cell Mol Med. 2020 May 4;24(12):6750–61. doi: 10.1111/jcmm.15326 (PMC7299728; doi:10.1111/jcmm.15326)

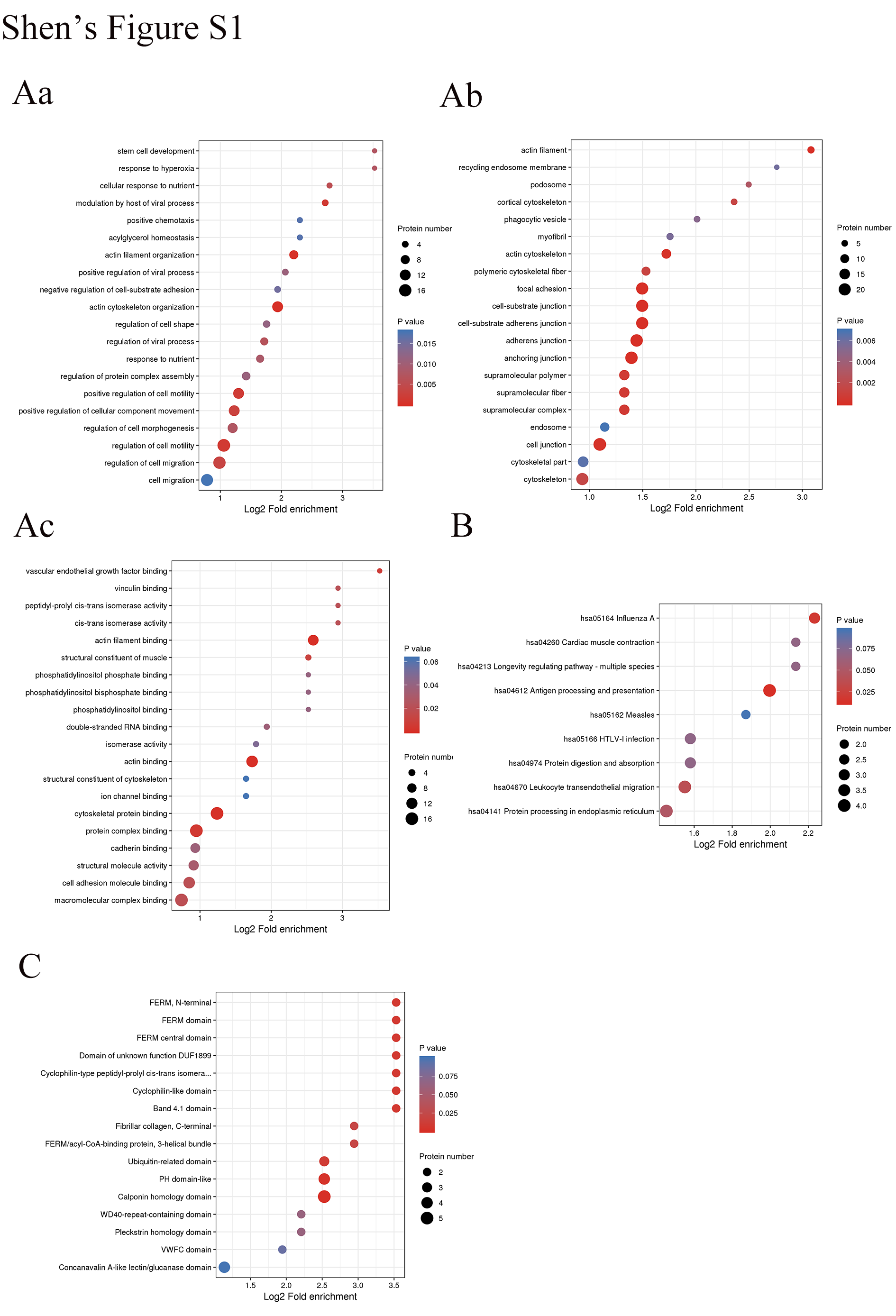

Supplement: Supplementary file 1 — Fig S1 [file JCMM-24-6750-s001.tif]
