# Supplementary material for: Serum proteome profiling reveals SOX3 as a candidate prognostic marker for gastric cancer
Source: J Cell Mol Med. 2020 May 4;24(12):6750–61. doi: 10.1111/jcmm.15326 (PMC7299728; doi:10.1111/jcmm.15326)
Supplement: Supplementary file 2 — Table S1 [file JCMM-24-6750-s002.docx]

**Supplementary Table S1. Correlation between SOX3 levels in tumor tissues and clinicopathological features of gastric cancer patients (TCGA database).**

| Clinicopathological features | N | Sox3 levels in tumor tissues | | *P*-value |
| --- | --- | --- | --- | --- |
|  |  | high cases (%) | low cases (%) |  |
| Age (years) |  |  |  | 0.027 |
| <60 | 105 | 20 (19.0) | 85 (80.9) |  |
| ≥60 | 199 | 20 (10.1) | 179 (89.9) |  |
| Gender |  |  |  | 0.816 |
| male | 195 | 25 (12.8) | 170 (87.2) |  |
| female | 109 | 15 (13.8) | 94 (86.2) |  |
| Tumor location |  |  |  | 0.683 |
| upper third | 74 | 10 (13.5) | 64 (86.5) |  |
| middle third | 108 | 11 (10.2) | 97 (89.8) |  |
| lower third | 113 | 18 (15.9) | 95 (84.1) |  |
| Others | 9 | 1 (11.1) | 8 (88.9) |  |
| Lymph node metastasis |  |  |  | 0.835 |
| N0 | 102 | 14 (13.7) | 88 (86.3) |  |
| N1- N3 | 202 | 26 (12.9) | 176 (87.1) |  |
| Primary tumor invasion |  |  |  | 0.041 |
| T1 and T2 | 87 | 6 (6.9) | 81 (93.1) |  |
| T3 and T4 | 217 | 34 (15.6) | 183 (84.3) |  |
| Distant metastasis |  |  |  | 0.428 |
| M0 | 289 | 37 (12.8) | 252 (87.2) |  |
| M1 | 15 | 3 (20.0) | 12 (80.0) |  |
| pTNM stage |  |  |  | 0.426 |
| I/II | 147 | 17 (11.6) | 130 (88.4) |  |
| III/IV | 157 | 23 (14.6) | 134 (85.4) |  |
