# Supplementary material for: Serum proteome profiling reveals SOX3 as a candidate prognostic marker for gastric cancer
Source: J Cell Mol Med. 2020 May 4;24(12):6750–61. doi: 10.1111/jcmm.15326 (PMC7299728; doi:10.1111/jcmm.15326)
Supplement: Supplementary file 3 — Supplementary Material [file JCMM-24-6750-s003.docx]

**Supplementary Materials and Methods**

**Protein Extraction, Trypsin Digestion, TMT/iTRAQ Labeling, HPLC Fractionation**

The cellular debris of serum sample was removed by centrifugation at 12,000 g at 4 ℃ for 10 min. Then, the supernatant was transferred to a new centrifuge tube. The top 12 high abundance proteins were removed by Pierce™ Top 12 Abundant Protein Depletion Spin Columns Kit (Thermo Fisher). Subsequently, the protein concentration was determined with BCA kit (Thermo Fisher) according to the manufacturer’s instructions. For digestion, the protein solution was reduced with 5 mM dithiothreitol for 30 min at 56 ℃ and alkylated with 11 mM iodoacetamide for 15 min at room temperature in darkness. The protein sample was then diluted by adding 100 mM TEAB to urea concentration less than 2M. Then, trypsin was added at 1:50 trypsin-to-protein mass ratio for the first digestion overnight and 1:100 trypsin-to-protein mass ratio for a additional 4 h digestion. After trypsin digestion, peptide was desalted by Strata X C18 SPE column (Phenomenex) and vacuum-dried. Peptide was reconstituted in 0.5 M TEAB and processed according to the manufacturer’s protocol for TMT kit/iTRAQ kit (Thermo Fisher). One unit of TMT/iTRAQ reagent were thawed and reconstituted in acetonitrile. The peptide mixtures were then incubated for 2 h at room temperature and pooled, desalted and dried by vacuum centrifugation. The tryptic peptides were fractionated into fractions by high pH reverse-phase HPLC (Agilent Technologies) using Agilent 300Extend C18 column (5 μm particles, 4.6 mm ID, 250 mm length). Briefly, peptides 37 / 40 were first separated with a gradient of 8% to 32% acetonitrile (pH 9.0) over 60 min into 60 fractions. Then, the peptides were combined into 18 fractions and dried by vacuum centrifuging.

**LC-MS/MS analysis**

The tryptic peptides were dissolved in 0.1% formic acid (solvent A), directly loaded onto a home-made reversed-phase analytical column (15-cm length, 75 μm i.d.). The gradient was comprised of an increase from 6% to 23% solvent B (0.1% formic acid in 98% acetonitrile) over 26 min, 23% to 35% in 8 min and climbing to 80% in 3 min then holding at 80% for the last 3 min, all at a constant flow rate of 400 nL/min on an EASY-nLC 1000 UPLC system. The peptides were subjected to NSI source followed by tandem mass spectrometry (MS/MS) in Q ExactiveTM Plus (Thermo) coupled online to the UPLC. The electrospray voltage applied was 2.0 kV. The m/z scan range was 350 to 1800 for full scan, and intact peptides were detected in the Orbitrap at a resolution of 70,000. Peptides were then selected for MS/MS using NCE setting as 28 and the fragments were detected in the Orbitrap at a resolution of 17,500. A datadependent procedure that alternated between one MS scan followed by 20 MS/MS scans with 15.0 s dynamic exclusion. Automatic gain control (AGC) was set at 5E4. Fixed first mass was set as 100 m/z.

**Spheroid cell invasion assay**

Spheroid cell invasion assay was carried out using 96 Well 3D Spheroid BME Cell Invasion Reagent Kit (Trevigen, MD, USA). Culture cells per manufacturer’s recommendation, and adherent cells should be cultured to no more than 80% confluence. 10× Spheroid Formation ECM was thawed on ice for 2 h or overnight in a 4 ℃ refrigerator. Harvested cells were resuspended in 1× Spheroid Formation ECM, and centrifuged at 200 g for 3 min at room temperature in a swinging bucket rotor. Then, cells were incubated at 37 ℃ in a tissue culture incubator for 72 h to promote spheroid formation. Invasion Matrix was thawed on ice for 4 h or overnight in a 4 ℃ refrigerator. 3D Culture Qualified 96 Well Spheroid Formation Plate was placed on ice in refrigerator for 15 min to cool wells. Working on ice, 50 µl of Invasion Matrix was added per well of the 3D Culture Qualified 96 Well Spheroid Formation Plate, and plate was centrifuged at 300 g at 4 ℃ for 5 min in a swinging bucket rotor to eliminate bubbles and position spheroids within the Invasion Matrix towards the middle of the well. Plate was transferred to a tissue culture incubator set at 37 ℃ for 1h to promote gel formation. One h later, 100 µl of cell culture medium containing chemoattractant and invasion modulating compounds was added, and was incubated at 37 ℃ in a tissue culture incubator for 3 to 6 d. The spheroid in each well was photographed every 24 h using the 4× objective. Images were analyzed to evaluate 3D culture cell invasion with Image J (<http://rsb.info.nih.gov/ij/>).
